# Bevacizumab-Based Therapies in Malignant Tumors—Real-World Data on Effectiveness, Safety, and Cost

**DOI:** 10.3390/cancers16142590

**Published:** 2024-07-19

**Authors:** Elena Chitoran, Vlad Rotaru, Sinziana-Octavia Ionescu, Aisa Gelal, Cristina-Mirela Capsa, Roxana-Elena Bohiltea, Madalina-Nicoleta Mitroiu, Dragos Serban, Giuseppe Gullo, Daniela-Cristina Stefan, Laurentiu Simion

**Affiliations:** 1 Medicine School, “Carol Davila” University of Medicine and Pharmacy, 050474 Bucharest, Romania; 2 General Surgery and Surgical Oncology Department I, Bucharest Institute of Oncology “Prof. Dr. Al. Trestioreanu”, 022328 Bucharest, Romania; 3 Radiology Department, Bucharest Institute of Oncology “Prof. Dr. Al. Trestioreanu”, 022328 Bucharest, Romania; 4 Obstetrics and Gynecology Department, “Filantropia” Clinical Hospital, 011132 Bucharest, Romania; 5 Surgery Department 4, Bucharest University Emergency Hospital, 050098 Bucharest, Romania; 6 Department of Obstetrics and Gynecology, Villa Sofia Cervello Hospital, University of Palermo, 90146 Palermo, Italy

**Keywords:** Bevacizumab, Avastin, angiogenesis inhibitors, VEGF, oncologic outcomes, survival, therapy-specific adverse effects, real-world experience, cancer metabolism, targeting metabolic vulnerabilities, recombinant humanized monoclonal antibody

## Abstract

**Simple Summary:**

Given the wide usage of Bevacizumab in current oncological practice, it is very important to compare the “real-world” results to those obtained in controlled clinical trials. This study aims to describe the clinical experience of using Bevacizumab in a large cohort of cancer patients in “non-controlled real-world” conditions regarding effectiveness, safety, and cost of therapy. For this purpose, we conducted an open, observational, retrospective study involving all patients treated for solid malignant tumors in the Bucharest Institute of Oncology with “Prof. Dr. Al. Trestioreanu” with Bevacizumab-based systemic therapy, between 2017 and 2021. Bevacizumab re-mains a high-cost therapy, but it can add to clinical benefits (like overall survival, progression-free survival, and response rate) when used in conjunction with standard chemotherapy. Similar results as those presented in various controlled trials are observable even on unselected cohorts of patients in the uncontrolled conditions of “real-world” oncological practice.

**Abstract:**

Overall, it is estimated that more than 3,500,000 patients have received Bevacizumab as part of systemic oncologic treatment. Bevacizumab and its biosimilars are currently marketed in over 130 countries. Given the wide usage of Bevacizumab in current oncological practice, it is very important to compare the “real-world” results to those obtained in controlled clinical trials. This study aims to describe the clinical experience of using Bevacizumab in a large cohort of cancer patients in “non-controlled real-world” conditions with regard to effectiveness, safety, and cost of therapy. Methods: For this purpose, we conducted an open, observational, retrospective study involving all patients treated for solid malignant tumors in the Bucharest Institute of Oncology with “Prof. Dr. Al. Trestioreanu” with Bevacizumab-based systemic therapy, between 2017 and 2021. Results: The study consisted of 657 treatment episodes in 625 patients (F/B = 1.62/1, with a median age of 57.6 years) which were treated for malignant tumors (majority colorectal, non-small cell lung, ovarian, and breast cancer). First-line treatment was administered in 229 patients, and the rest received Bevacizumab as second or subsequent lines of treatment. The overall response rate to Bevacizumab-based therapies was around 60–65% across all indication except for subsequent treatment lines in colorectal and ovarian cancers, where lower values were recorded (27.1%, and 31.5% respectively). Median PFS for the entire cohort was 8.2 months (95% CI 6.8–9.6), and the median OS was 13.2 months (95% CI 11.5–14.9). Usual bevacizumab-related toxicities were observed, including bleeding, hypertension, wound-healing complications, gastrointestinal perforation, other types of fistulas, septic complications, and thromboembolic events. Although the clinical benefits are undeniable, the addition of Bevacizumab to standard chemotherapy increased the overall treatment cost by 213%. Conclusions: Bevacizumab remains a high-cost therapy, but it can add to clinical benefits (like overall survival, progression-free survival, and response rate) when used in conjunction with standard chemotherapy. Similar results as those presented in various controlled trials are observable even on unselected cohorts of patients in the uncontrolled conditions of “real-world” oncological practice. Off-label usage is encountered in clinical practice, and this aspect should be monitored given the potential adverse effects of the therapy.

## 1. Introduction

The tumorigenesis of solid cancers is a multifactorial process in which genetic and epigenetic mechanisms cause an imbalance of tumor suppressor genes and oncogenes, resulting in anarchic, rapid cellular growth. As the hyperplastic cellular mass reaches a critical size, the supply of oxygen and nutrients or the removal of waste products, as functions of the increasingly larger distance from the nearest emerging blood vessel, can no longer be supported by the microenvironment of the peritumoral tissue of origin. Under these conditions, to meet the continuously increasing metabolic demands, solid tumors must develop their own circulation [1,2]. The newly formed vessels originate from the pre-existing vessels of the normal peritumoral tissues and are the result of the alteration of the balance between positive and negative regulators of vascular growth (a process also known as the “angiogenic switch”) [2,3,4,5,6]. Although the “angiogenic switch” is a relatively discreet process, it is a limiting stage of tumorigenesis. Neo angiogenesis accompanies tumor progression from the avascular hyperplasia phase to the vascularized neoplastic phase, providing support for tumor expansion and metastasis [1,7,8]. The growth of solid tumors beyond 0.2–2 mm has been proven, in experimental models, to depend on angiogenesis [9]. The most important and well-known positive molecular regulators of tumor neo angiogenesis are members of the VEGF (vascular endothelial growth factor) family, which are secreted in the form of dimeric glycoproteins with a molecular weight of 36–46 kilodaltons and act through tyrosine kinase (TK) receptors and co-receptors (neuropilins NRP-1 and NRP-2). The VEGF family in mammals consists of five members, designated as VEGF-A, B, C, D, and Placental Growth Factor (PlGF) [10,11], and three receptors belonging to class IV of the tyrosine kinase receptors (TKR)-VEGFR-1 (fms-like TK1, Flt1), VEGFR-2 (Kinase insert domain receptor–KDR, Flk1), and VEGFR-3 (fms-like TK4, Flt4) [10]. The binding of VEGF ligands to receptors leads to their autophosphorylation, triggering a downstream phosphorylation cascade, thereby initiating an intracellular signaling pathway that controls a series of mechanisms involved in neo angiogenesis and inflammation. The overexpression of the VEGF gene is regulated by various cytokines (such as nitric oxide, IL-1, IL-6), growth factors (platelet-derived growth factor, keratinocyte growth factor, epidermal growth factor–EGF, tumor necrosis factor alpha–TNF-α, fibroblast growth factor-4), and hormones (insulin, corticotropin, thyrotropin, steroid hormones) [12].

In accordance with its role in tumor neo angiogenesis, VEGF expression is also stimulated by common genetic events leading to malignant transformation, such as the loss of tumor suppressor genes (p53) and the activation of oncogenes (ras, v-src, Her2). Additionally, under hypoxic conditions, VEGF expression is intensely stimulated through multiple mechanisms [13]. The activation of VEGF transcription is mediated by the hypoxia-inducible factor-1 (HIF-1) dimeric protein, consisting of two subunits (alpha and beta). The alpha subunit of HIF-1 is unstable under normal levels of oxygen but becomes stable in hypoxia. Hypoxia also stabilizes VEGF mRNA. VEGF overexpression has been correlated with lymphatic invasion and metastasis. Patients with high levels of VEGF expression have poorer survival rates compared to those with low or negative levels. Preoperative VEGF values are correlated with advanced neoplastic disease [14,15]. VEGF levels are predictive of metastatic potential, independent of nodal status and adjuvant chemotherapy, with a positive predictive value of 73% [16].

In the context of the aforementioned points, Judah Folkman’s postulates, which first suggested in 1971 that tumor angiogenesis could be a potential therapeutic target [2], have shifted research in neoplastic disease treatment from classical tumor cell-centered therapies to approaches targeting the inhibition of pathological angiogenesis. This shift has led to the emergence of a new field in oncology [7,17,18,19], with the blockade of the VEGF/VEGFR signaling pathway becoming a primary target in the development of new therapeutic agents for solid tumors. Numerous drugs with antiangiogenic activity have been developed and approved by international competent authorities for use in the treatment of neoplastic diseases. However, anti-neoplastic therapies have focused solely on inhibiting the formation/growth of new blood vessels and/or destroying pre-existing vessels remain suboptimal, showing limited clinical efficacy [20,21,22]. Therapies developed to block the VEGF/VEGFR axis are significantly influenced by the specificities of neo angiogenesis in each tumor type. Thus, due to the heterogeneity of the angiogenic response, blocking the VEGF/VEGFR axis can result in therapeutic responses of varying intensities and durations in different organs [23]. Moreover, the success of targeting the heterogeneous population of vessels in the tumor stroma is strongly influenced by the dependency of the VEGF effect on the state of cellular maturation, pericyte density, and the tumor stromal microenvironment [24,25,26]. Consequently, pancreatic carcinoma is often refractory to anti-angiogenic treatment, as is hepatocarcinoma, where therapeutic effects are limited despite the tumor’s hypervascularization [17,27]. Therapy with angiogenesis inhibitors can paradoxically lead to the selection of cellular clones adapted to survive in a hypoxic environment, especially in the tumor center. Inhibition of a specific pro-angiogenic factor may lead to compensatory reactions (with the activation of secretion and release of alternative stimulating factors from selected cells) [28,29,30,31,32,33,34] or the recruitment of other cell types with pro-inflammatory/pro-angiogenic phenotypes [35]. Thus, adaptive resistance mechanisms and compensatory refractoriness can severely limit the efficacy of single-agent anti-angiogenic therapeutic modalities.

Bevacizumab (Avastin^TM^) is an antineoplastic, recombinant humanized monoclonal antibody belonging to the IgG1 class, produced by DNA technology, containing 93% human sequences and 7% murine sequences, with a molecular structure of 149 kilo-Daltons and composed of 1320 amino acids-C6638 H10160 N1720 O2108 S44 [36]. The monoclonal antibody has a structure comprising two heavy chains (H-heavy, each containing 453 amino acids) and two light chains (L-light, each containing 214 amino acids), linked by disulfide bonds. Both types of chains contain variable regions (V_H_ and V_L_) and constant regions (C_H_ and C_L_). Bevacizumab and its biosimilars binds selectively and with high affinity to all VEGF isoforms, thereby neutralizing the biological action of VEGF from sterically binding to its receptors VEGFR-1 (Flt1) and VEGFR-2 (KDR) on the surface of endothelial cells, thus blocking VEGF-mediated pro-angiogenetic intracellular signaling pathways. The blockade has the effect of decreasing the vasculature of solid tumors and decreasing microvascular permeability, resulting in the inhibition of tumor growth and decreased metastatic potential. Due to the very low or undetectable expression of VEGF receptors in normal tissues (except renal glomeruli), the use of Bevacizumab produces a relatively specific inhibition of neoplastic neo angiogenesis. By decreasing interstitial pressure and increasing vascular permeability, Bevacizumab can increase tissue distribution of other therapeutic agents [37,38], potentiating their action, making concomitant administration rational.

Bevacizumab, one of the first therapies targeting the tumor microenvironment [39], added to standard chemotherapy, has led to an effective therapeutic option for a series of advanced solid tumors, which, before the development of targeted therapies, were known to have a poor prognosis. Tumor microenvironments play a vital role in cancer therapy [40]. Since its first approval for usage in humans with colorectal carcinoma [41,42,43,44,45,46,47], Bevacizumab has been approved for both first and subsequent lines of therapy (including maintenance), usually in combination with various chemotherapeutic agents, for metastatic, locally advanced unresectable or recurrent cancer–breast [48,49], ovarian [50,51,52,53,54,55,56], cervical [57,58], pulmonary (non-small cell) [59], renal carcinoma [60,61], primary peritoneal, or glioblastoma [62,63,64,65]. There is, however, a lack of consensus between the two main regulatory agencies: European Medicine Agency (EMA)–which has not approved Bevacizumab for Glioblastoma treatment [66]–and the Food and Drug Administration (FDA)–which has retired the approval of Bevacizumab for metastatic breast cancer [67,68,69].

Deficiencies in the approval process of anti-tumoral agents may add to the confusion about the actual efficacy of Bevacizumab and its biosimilars. Avastin (the original Bevacizumab molecule subject to FDA approval) has been approved following the results of clinical trial conducted by the manufacturer, which used surrogate endpoints (like progression-free survival) to evaluate clinical benefits and efficacy of the drug rather than standard clinical outcomes (like overall survival). The definition of a clinical outcome, according to the FDA, is “a direct measure of benefit of an intervention in a trial” and a surrogate endpoint is a “predictive substitute for clinical outcomes”. The practice of using surrogate endpoints is frequently used for approval of anti-tumoral drugs in solid tumor therapy [70,71]. This is done for a more rapid approval of drugs that seem to have clinical effects in patients with severe pathologies for which there is no current optimal control therapy. However, it is important to remember that 57% of all anti-tumoral agents approved using surrogate endpoints, and the efficacy was not confirmed when the mature overall survival data from the trials became available [72]. Several studies have already highlighted the fact that progression-free survival is a suboptimal replacement for the standard clinical outcome (OS) [73,74,75,76]. Avastin failed to prove overall survival benefits in patient with metastatic breast cancer when final trial data were available, and as a result, the FDA retired the approval of the drug for this indication [67,68,69]. Likewise, biosimilars are approved after a rapid process which does not include all the standard steps; the manufactures just need to prove bio similarity of the molecules and safety profile, with the clinical benefits being extrapolated from the already approved parent drug. In this context, it is of paramount importance to confirm the results of trials describing the effect of Bevacizumab and its biosimilars in clinical practice.

Although other anti-angiogenic drugs have been developed—mostly small molecule multi-kinase inhibitors—that block the angiogenic pathway of VEGF and/or other pro-angiogenic signaling pathways, Bevacizumab, the oldest anti-angiogenetic drug, remains the most widely used [11,77,78]. More than 37,000 patients were treated with Bevacizumab during clinical trials [79]. Overall, it is estimated that more than 3,500,000 patients have received Bevacizumab as part of systemic oncology treatment [36]. Bevacizumab is currently marketed in over 130 countries. Given the wide usage of Bevacizumab in current oncological practice, it is very important to compare the “real-world “results to those obtained in controlled clinical trials.

This study aims to describe the clinical experience of using Bevacizumab in a large cohort of cancer patients in non-controlled “real-world” conditions regarding effectiveness, safety, and cost of therapy. In the era, where new therapeutic approaches are considered every day with the sole purpose of improving quality of life of cancer patients and patient-reported outcomes [80,81,82,83], it is necessary to conduct these “real-world” evaluations, proving/disproving trial data.

## 2. Materials and Methods

### 2.1. Patient Selection

This study was conducted in accordance with the Good Clinical Practice guidelines and the Declaration of Helsinki and was approved by the Institutional Revied Board of Bucharest Institute of Oncology “Prof. Dr. Al. Trestioreanu”, Romania (approval no.24855/24 November 2022). The method used was an open, observational, retrospective, real-world study carried out on patients treated for solid malignant tumors in the Bucharest Institute of Oncology “Prof. Dr. Al. Trestioreanu” (one of the three major regional Oncology Centers in the country that ensure cancer treatment for a population of 19 million). For this study all patients receiving Bevacizumab-based systemic therapy, between 1 January 2017 and 31 December 2021.

### 2.2. Data Extraction

As this research was intended to evaluate the usual clinical practice and the real-world experience of using this medication, and all data were extracted from medical records and electronic databases, no incentives of any kind were given to either participating patients or physicians, and no additional evaluations were required except those already in the medical files. No exclusion criteria were stated, and all medical decisions about treatment, doses, regimens, or follow-up were made by clinicians without any limitation or influence. All patients admitted to this hospital agree, by signing a consent form, to the anonymous usage of medical records and investigation results for scholarly analysis, educational purposes, and preparing and publishing scientific papers, and no additional consent was needed unless the patient had retired his/her consent. However, the hospital management approved and gave permission for retrieving data from internal database and/or medical records.

Patients receiving Bevacizumab were identified by the pharmacy consumption tables. If a patient received more than one line of therapy containing Bevacizumab, each line of treatment was considered a separate record and analyzed as such. Similarly, if a patient received Bevacizumab for more than one neoplastic disease, each indication was considered a separate record. Specific case data were obtained from the patients charts and electronic records and by consulting the results of paraclinical, imaging, histopathological, and genetic results.

Data collected included demographics (age, gender), comorbidities and relevant medical history, cancer history (tumor origin, histology, immunohistochemistry, genetic characteristics, metastatic sites, dates of diagnostic, beginning of treatment, progression, death), and treatment aspects (line of treatment, combination therapies, dosage, duration of treatment, adverse events, response to treatment, cost of treatment, hospitalization). Costs of anticancer drugs were assessed from the pharmacy records (mentioning price/unit and dosage of unit). The hospitalization-associated costs were the direct costs allocated to the Oncology Department by the Internal Analytical Accounting System.

Effectiveness of Bevacizumab treatment was measured by analyzing progression-free survival (PFS)—defined as the time between the start of therapy and first record of disease progression either assessed by clinician or proved by clinical investigations, including imaging, or until death—and overall survival (OS) was defined as the time between the start of therapy and recorded death of patient or censoring. Response rates were evaluated according to RECIST criteria [84]. In December 2023, all patients still in follow-up but without any recorded event were censored. Adverse events were classified according to National Cancer Institute-Common Terminology Criteria for Adverse Events [85].

### 2.3. Statistical Analysis

Categorical data were analyzed by counts and percentages; by calculating means, standard deviations, medians, and ranges; and by comparing different groups using the Chi square test. Continuous data were analyzed as means, and standard deviations and comparison between groups were performed using Student *t*-tests. Oncological results, like overall survival (OS) and progression-free survival rate (PFS), were analyzed, and a comparison between various subsets of patients was performed. Cox proportional hazard regression modelling was used to evaluate potential factors influencing oncologic results. A *p*-value lower than 0.05 was considered statistically significant.

## 3. Results

### 3.1. Population Characteristics

The study included 657 episodes of treatment with Bevacizumab or biosimilars, corresponding to 625 unique patients: 27 patients were on two treatment lines containing Bevacizumab, 1 patient received three, and 3 patients received Bevacizumab-based therapies for two distinct cancers (2 patients had colorectal and breast carcinomas, and 1 had breast and uterine cancers). The F/M ratio was 1.62/1. Overall, 62.56% of patients were ≥65 years old. Among the most frequent comorbidities, we observed arterial hypertension (36.32%), diabetes mellitus (14.4%), chronic pulmonary disease (6.88%), and other cancers (1.12%). However, all comorbidities were controlled by therapy and did not hinder the administration of systemic chemotherapy or Bevacizumab. Study inclusion was not limited by indication or tumor origin, thus providing a “real-world” heterogenous, uncontrolled population. Of course, this also represents one of the limitations of the study because the composition if the study cohort is highly influenced by the addressability of our hospital, which differs from that of other hospitals, creating the possibility for the sample to not be highly representative for all “real-world” oncological practice.

Our cohort included mostly patients with colorectal cancers (almost 60% of treatment episodes), but also included advanced ovarian cancer (20.09%), breast cancer (8.07%), non-squamous-cell lung cancer (7.15%), cervical cancer (4.72%), and other primary origins (vagina, vulva, peritoneal, central nervous system)–but this last category included a very low number of patients (eight treatment episodes). Out of the 625 patients, 588 had metastatic lesions. As for metastatic sites, patients may have had multiple localizations. More than half of all patients had more than one metastatic site (331 patients, 52.96%). Our study also included 37 patients with no signs of systemic disease who had been treated for recurrent/locally advanced unresectable malignant tumors. The most frequent metastatic sites included liver—391 (62.56%), lung—344 (55.04%), and peritoneum—156 (24.96%).

First-line treatment was administered in 229 patients (185 colorectal, 18 breast, 26 NSCLC), and subsequent lines in 428 patients. Bevacizumab or its biosimilars (Mvasi, Alymsys/Oyavas) were administered in conjunction with standard chemotherapy as deemed necessary by the patient’s oncologist with no external interference. Out of the 657 treatment episodes, in 509, Avastin was used, and in the rest, biosimilars were administered. All patients treated before 2020 received Avastin. As a result of EMA approval of Mvasi and Alymsys/Oyavas and the subsequent introduction of these biosimilars in the national oncology program (which allows for the patient to benefit from the drugs without pay on basic health insurance, the costs being supported by the state), these drugs were included in clinical practice from 2020 and 2021, respectively. Patient and treatment characteristics are summarized in Table 1.

### 3.2. Response to Treatment

Out of 657 treatment episodes, only 610 were taken into consideration when we evaluated responses (47 episodes did not have data on the physician-assessed response or supportive imaging investigations). The overall response rate across all indications (term encompassing all cases with noticeable response to therapy–either partial or complete) was 50.82% (310 patients). The overall response rate was calculated by adding all patients with noticeable shrinkage of tumor(s) either assessed by clinical examination or by imaging measuring. A partial response was defined as shrinkage of tumor upon clinical/imaging examination after therapy, but with evident residual tumor. A complete response was defined as no clinical/imaging evidence of tumor after therapy. In 29 patients (4.75%), the response was considered complete. The overall response rate to Bevacizumab-based therapies was between around 60 and 65% across all indication, except for subsequent treatment lines in colorectal and ovarian cancers, where lower values were recorded (27.1% and 31.5%, respectively). Clinical benefits–defined as the amelioration of symptoms and laboratory findings–were observed in 416 patients (66.56%), and the clinical benefits were not necessarily associated with tumor shrinkage (amelioration of laboratory findings were present even in some patients with no apparent tumoral shrinkage on clinical/imaging examination). Median PFS for the entire study cohort was 8.2 months (95% CI 6.8–9.6), and the median OS was 13.2 months (95% CI 11.5–14.9). Progression-free survival (PFS) was defined as the time between the start of therapy and first record of disease progression, either assessed by clinician or proved by clinical investigations including imaging, and overall survival (OS) was defined as the time between the start of therapy and recorded death of patient or censoring. No significant differences of ORR, PFS, or OS between patients under and over 65 years was observed. Median PFS median OS and the response rate by tumor type and treatment line are summarized in Table 2.

### 3.3. Safety Analysis–Adverse Events

**Figure 1:**
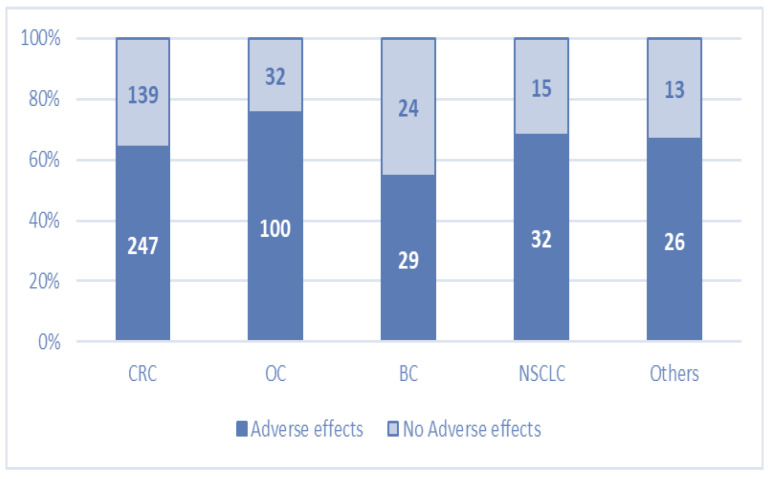
Adverse events of all grades per cancer type: CRC—colorectal cancer; OC—ovarian cancer; BC—breast cancer; NSCLC—non-small-cell lung cancer.

Bevacizumab-related toxicity of any grade was reported in 434 patients, and the frequency of adverse events varied from 55% in patients with BC to 76% in patients with OC (Figure 1). In this figure, we can see that the percentage of patients experiencing at least one adverse effect after therapy was larger than patients with no adverse effects across all indications. This is due to the fact that this figure includes all adverse effects, including grade 1–2 gastrointestinal effects (such as nausea and diarrhea) or minimum blood loss (through gums or nasal pathways). The usual minimal/mild treatment-related adverse events were observed in our cohort, which did not affect the continuation of therapy in any way and did not cause an additional need for medical care. Severe adverse reactions were fewer and required the cessation of Bevacizumab therapy. Table 3 summarizes both the adverse effects of any grade occurring with a frequency >5% of patients and adverse effects that determined discontinuation of treatment. Observed toxicity included bleeding (most minor/mild nasal or gum bleeding), hypertension, proteinuria, wound-healing deficiencies, gastrointestinal perforation, other types of perforation/fistular complications, septic complications, thromboembolic events, abdominal pain, diarrhea, nauseas/vomiting, and fatigue. Treatment was ceased due to adverse events in 81 patients (12.33%). Mortality within this cohort consisted of three patients (thromboembolic or septic complications).

### 3.4. Cost of Treatment

In our study, the population of patients received a median of 13 (range 1–76) doses of Bevacizumab in one treatment episode with a mean quantity of 657 mg/dose. The total acquisition cost for the necessary quantity of Bevacizumab/biosimilars between 2017 and 2021 was EUR 25.5 million, with a median treatment episode cost of EUR 38.812. The cost of Bevacizumab represented 68% of total treatment costs. Adding Bevacizumab to standard chemotherapy increases the overall cost of treatment by 213%.

Overall, 126 patients required hospitalization or extension of hospital stay because of Bevacizumab-induced toxicity of 2–24 days; there was a statistically significant association between grade 3–5 adverse events and hospitalization (*p*-value 0.002). Beyond the added price of the actual drug, Bevacizumab determined an increased cost of hospitalization-associated costs when severe adverse events occurred. The hospitalization-associated costs were the direct costs allocated to the Oncology Department by the Internal Analytical Accounting System and included fixed costs (housing, utilities) and variable expenditures, such as costs of laboratory analysis and other diagnostic procedures, cost of medication (both antineoplastic and comedication), and instruments, medical devices, consumables). The increased costs generated are of great concern to medical management all over the world [86].

Among the complications observed, some required surgical procedures (17 cases); such cases were associated with a hospitalization length of 10–32 days and an increase of hospitalization associated costs of 150–495% when compared to the standard cost of therapy in patients treated that did not develop severe adverse effects. The surgical procedures can be very challenging in the context of oncologic patients with a heavy personal history of surgery and radiotherapy [87,88].

Off-label usage was encountered in only three cases (advanced unresectable hepatocarcinoma–two cases and metastatic pancreatic cancer–one case).

## 4. Discussion

All anti-cancer drugs are approved by the regulatory agencies (EMA and FDA) as the result of clinical trials that are usually ordered and financed by the manufacturer of said drugs. The results of such trials, conducted under very rigorous conditions on selected groups of patients, may yield results which differ and are not representative of the whole population requiring treatment in the community. Moreover, the approval of many anti-cancer drugs follows an accelerated procedure. Bevacizumab (Avastin^TM^) was first approved by the FDA for use in patients with metastatic breast cancer in February 2008 through the accelerated approval program. A drug can be approved through this rapid mechanism based on a biomarker or surrogate endpoint for overall survival (such as dimensional involution of the tumor under therapy) that suggests an important clinical benefit for patients (based on immature data from ongoing clinical trials). Accelerated approval may be granted for drugs that treat severe conditions for which there are currently no effective treatments. However, the methodological rules of this rapid approval procedure require the presentation of the results of subsequent trials that confirm significant clinical benefits such as increased overall survival [67], and it is the manufacturer’s responsibility to prove these benefits. If they fail to do so, the approval can be retired; –the FDA has, in fact, withdrawn the approval of the breast cancer indication for Avastin^TM^. Although not as rigorous as randomized controlled trial, “real-world” studies offer the possibility of verifying experimental data on non-selected populations (arguably more representative for the general population of oncologic patients), thereby expanding existing data on the efficiency and safety of Bevacizumab.

The efficacy of treatment was examined, and we found an overall response rate across all indications of 50.82%, with lower response rates of subsequent treatment lines in colorectal and ovarian cancers (27.1% and 31.5%, respectively). Median PFS for the entire study cohort was 8.2 months, and the median OS was 13.2 months. No significant differences of ORR, PFS, or OS between patients under and over 65 years were observed–probably due to already proven good tolerability of Bevacizumab by elderly patients [89,90]. The results are similar to those presented and controlled clinical trials [55]. The median PFS observed when Bevacizumab was used as a first line of treatment for colorectal carcinomas was, in fact, higher than those reported previously [91]. Our study cohort had, from the beginning, several unfavorable prognostic factors (such as multiple metastatic sites in the same patient or multiple previous treatment lines), yet the oncological results were not substantially different from those reported by controlled clinical trials. Table 4 summarizes the comparison between our results and the results of the controlled clinical trial on which approval for the clinical usage of Bevacizumab and biosimilars was obtained.

Similarly, no significant differences were observed in the proportion of patients reporting adverse effects. Usual bevacizumab-related toxicities were observed, including bleeding, hypertension, wound-healing complications, gastrointestinal perforation, other types of fistulas, septic complications, and thromboembolic events. There are studies which have stated that the addition of Bevacizumab to standard chemotherapy or immunotherapy was associated with an increase in treatment-related mortality [92]; our study did not show similar findings, with the observed mortality being 0.45%.

Although the clinical benefits are undeniable, the addition of Bevacizumab to standard chemotherapy increases the overall treatment cost by 213%, which raises questions about the cost-effectiveness of the drug. In fact, the addition of Bevacizumab (Avastin^TM^) to standard therapy is credited with PFS benefits of a few months, and there are conflicting data about whether it has any OS benefits [93,94]. When adding the risk of serious drug-related toxicity, the justification of a price tag of around EUR 40 thousand per patient becomes problematic.

Our data reflected the standard chemotherapy usage patterns in Europe–as a result we encountered little off-label usage (although including many patients with metastatic breast cancer, their treatment is not considered off-label because the European Medicine Agency has maintained the approval of Bevacizumab for this indication). Off-label usage was associated with young age (<50 years) and high economic status of the patient.

The limitations of the studies are several and are related to the open observational design. In order to mitigate the bias, and because this research was intended to evaluate the usual clinical practice and the “real-world” experience of using this medication and all data were extracted from medical files or electronic databases, no incentives of any kind were given to either participating patients or physicians, and no additional evaluations were required except those already in the medical records. No exclusion criteria were stated, and all medical decisions about treatment, doses, regimens, or follow-up were made by clinicians without any limitation or influence. Although patient identification was done by searching the electronic hospital database, we cannot exclude a selection bias due to possible data loss by cybernetic attacks which occurred prior to us conducting the search. Since response to treatment was, in part, evaluated by physician, there is a possible evaluation bias. Similarly, the follow-ups were decided by each oncologist and the periods between follow-up sessions were not uniform, there is a possibility of over-estimation of PFS (as a result of longer periods between follow-up sessions). Other limitations include non-uniform therapeutic regimens, dosing, and scheduling (which make a global analysis very difficult if not impossible) and a limited number of patients with certain kinds of tumors (in these groups, an extrapolation of outcomes was impossible, and the patients were grouped together). Moreover, the results may be influenced by the composition of our study cohort (the mix of patients may not be representative for the entire population treated with Bevacizumab or biosimilars for solid tumors) and by the fact that, in Romania, most patients are diagnosed in advanced stages, thus limiting the therapeutic options and influencing overall results [95,96]. Given these limitations, it is rational to perform, in the future, a pooled analysis of all “real-world” cohorts available for a better evaluation of effectiveness and safety of Bevacizumab in solid malignant tumors, which may mitigate the inherent biases of observational studies.

**Table 4:** Comparison between our results and the results of primary controlled clinical trials which led to the approval for clinical usage of Bevacizumab by cancer type.

Study	N	Design	Population	Results/Findings	Biases/Discussion
**Colorectal cancer**					
Our study	379	Retrospective, Real-world data Unselected patients	mCRC 1L (N = 294) 2+ lines (N = 85)	1L: PFS 13.5 mo; OS 26.3 mo; RR 60.9% 2+ lines: PFS 6.2 mo; OS 9.3 mo; RR 27.1%	Selection bias Non-uniform therapeutic regimens, dosing and scheduling
AVF2107g [46,91,97,98]	923	Phase III, PL controlled, randomized Multicenter	mCRC 1L arm 1 (N = 411): IFL + PL arm 2 (N = 402): IFL + Bev 5 mg/kgc q2wk arm 3 (N = 110): FL + Bev 5 mg/kgc q2wk Enrollment in arm 3 was ceased prematurely due to safety concerns	OS: adding Bev to IFL improved OS from 15.6 to 20.3 months (*p* < 0.0001) PFS: adding Bev to IFL improved PFS from 7.06 to 10.35 months (*p* < 0.0001) RR: 44.8% in combined therapy QoL: no difference between arms in the time until deterioration (*p* = 0.5807)	Selection deficiencies Major breaches of study protocol in 39.9% of patients in arm 1 and in 49.5% of patients in arm 2
AVF0780g [43,45,91]	104	Phase II, randomized Multicenter, multidose, open-label	mCRC 1L arm 1 (N = 36): FL arm 2 (N = 35): Bev 5 mg/kgc q2wk + FL arm 3 (N = 33): 10 mg/kgc q2wk + FL	OS: addition of Bev to FL improves OS from 13.6 mo to 17.7 mo (arm 2) and 15.2 mo (arm 3)–results were not statistically significant (*p* > 0.05) PFS: addition of Bev 5 mg/kgc to FL improved PFS from 5.2 mo to 9.0 mo (*p* = 0.0049); 10 mg/kgc of Bev was not associated with improved PFS RR: arm 1–16.7%; arm 2–40% (*p* = 0.029); arm 3–24.2% (*p* = 0.43)	Multiple protocol deviations Small sample
AVF2192g [91]	209	Phase II, PL controlled, randomized	mCRC 1L, in patients who were not optimal candidates for irinotecan arm 1 (N = 105): FL + PL arm 2 (N = 104): FL + Bev 5 mg/kgc q2wk	OS: although an improvement was observed in arm 2, it was not significant PFS: addition of Bev to FL increased PFS from 5.5 to 9.2 mo (*p* = 0.0002) RR: no significant difference between arms was observed	NS
XELOX-1 (NO16966) [91,99,100]	1401	Phase III randomized, PL controlled, double-blind	mCRC 1L Bev 7.5 mg/kgc q3wk + XELOX or PL and Bev 5 mg/kgc q2wk + FOLFOX-4 or PL	Superiority of Bev-containing arms versus chemotherapy alone arms in the overall comparison was demonstrated in terms of PFS No significant difference in OS was observed by adding Bev to chemotherapy	NS
E3200 [41,91]	829	Phase III randomized, controlled, open-label	mCRC, 2nd-line, Bev-naïve patients Arm 1 (N = 292): FOLFOX-4 Arm 2 (N = 293): Bev 10 mg/kgc q2wk+ FOLFOX-4 Arm 3 (N = 244): Bev monotherapy	OS: addition of Bev to FOLFOX-4 increased OS from 10.8 to 13.0 mo (*p* = 0.0012) PFS: addition of Bev to FOLFOX-4 increased PFS from 4.5 to 7.5 mo (*p* < 0.0001) RR: arm 1–8.6%; arm 2–22.2% (*p* < 0.0001) No significant difference was observed in OS between arm 3 and arm 1 PFS and RR were inferior in arm 3 compared to arm 1	NS
ML18147 [91,101]	819	Phase III, randomized, controlled, open-label	2nd-line with previous Bev treatment following disease progression after 1L, arm 1 (N = 410): Bev 5.0 or 7.5 mg/kgc q2wk + fluoropyrimidine/irinotecan arm 2 (N = 409): fluoropyrimidine/oxaliplatin	OS: addition of Bev increased OS from 9.8 to 11.2 mo (*p* = 0.0062) PFS: addition of Bev increased PFS from 4.1 to 5.7 mo (*p* < 0.0001) RR: no significant difference was observed	NS
**Breast cancer**					
Our study	51	Retrospective, Real-world data Unselected patients	m/r BC 1L (N = 23) 2+ lines (N = 28)	1L: PFS 10.2 mo; OS 19.7 mo; RR 65.2% 2+ lines: PFS 8.1 mo; OS 15.6 mo; RR 60.7%	Selection bias Non-uniform therapeutic regimens, dosing, and scheduling
ECOG E2100 [91,102,103]	722	Phase III, open-label, randomized, active controlled, multicenter	m/r BC Arm 1 (N = 354): paclitaxel Arm 2 (N = 368): paclitaxel + Bev 10 mg/kgc q2wk	PFS: addition of Bev increased PFS from 5.8 to 11.4 mo (*p* < 0.0001) RR: arm 1–23.4%; arm 2–48.0% (*p* < 0.0001) No significant OS benefit was observed	NS
RIBON 1 (AVF3694g/BO20094) [91,104]	1237	Phase III, multicenter, randomized, PL-controlled	m/r BC 1L, Her2-negative patients chemotherapy (capecitabine or anthracycline/taxanes) + PL chemotherapy (capecitabine or anthracycline/taxanes) + Bev 15 mg/kgc q3wk	PFS: addition of Bev to capecitabine increased PFS from 6.2 to 9.8 mo (*p* = 0.0011) RR: addition of Bev to capecitabine increased RR from 23.6% to 35.4% (*p* = 0.0097) Similar results were observed when adding Bev to anthracyclines/taxanes No significant OS benefit was observed	Significant increase of adverse reactions in Bev arms
AVADO (BO17708) [90,91]	736	Phase III, randomized, PL-controlled, double blind	m/r BC 1L, Her2 negative patients arm 1: Docetaxel + PL arm 2: Docetaxel + Bev 7.5 mg/kgc arm 3: Docetaxel + Bev 15 mg/kgc	Addition of Bev 7.5 mg/kgc led to a 30% increase of PFS (HR = 0.70; CI95% 0.55–0.90) Addition of Bev 15 mg/kgc led to a 39% increase of PFS (HR = 0.62; CI95% 0.48–0.79) RR: arm 1–44%; arm 2–55%; arm 3–63% (increases in Bev arms were significant) OS has decreased in both Bev arms, but this result was not significant	The absolute improvement in PFS was, in fact, quite small–0,8 mo and, 0.88 mo respectively Significant increase of adverse reactions in Bev arms
**NSCLC**					
Our study	32	Retrospective, Real-world data Unselected patients	m/a/r NSCLC 1L (N = 27) 2+ lines (N = 5)	1L: PFS 7.4 mo; OS 12.6 mo; RR 66.7% 2+ lines: PFS 8.4 mo; OS 13.1 mo; RR 60%	Selection bias Non-uniform therapeutic regimens, dosing, and scheduling
ECOG E4599 [91,105,106]	878	Phase II, open-label, randomized, controlled, multicenter	a/m NSCLC arm 1 (N = 444): carboplatin/paclitaxel arm 2 (N = 434): carboplatin/paclitaxel + Bev 1 mg/kgc q3wk	OS: addition of Bev improved OS from 10.3 to 12.3 mo (HR 0.80, CI95% 0.69–0.93, *p* = 0.003) PFS: addition of Bev improved PFS from 4.8 to 6.4 mo (HR 0.65, CI95% 0.56–0.76, *p* < 0.0001) RR: addition of Bev improved RR from 12.9% to 29% (*p* < 0.0001)	In subgroup analysis, the OS benefits were less pronounced in patients with other histology than adenocarcinoma
AVAiL (BO17704) [91,107]	1043	Phase III, randomized, double bind PL-controlled	a NSCLC 1L arm 1 (N = 347): cisplatin/gemcitabine + PL arm 2 (N = 345): cisplatin/gemcitabine + Bev 7.5 mg/kgc q3wk arm 3 (N = 351): cisplatin/gemcitabine + Bev 15 mg/kgc q3wk	No significant OS benefits were observed in Bev arms PFS was improved in both Bev arms but only by an absolute difference of 0.6 mo (in arm 2) and 0.4 mo (in arm 3) RR: 20.1%-arm 1; 34.1%-arm 2 (*p* < 0.0001); 30.4%-arm 3 (*p* = 0.002)	NS
**Ovarian cancer**					
Our study	127	Retrospective, Real-world data Unselected patients	a/m/r OC	PFS 7.0 mo; OS 11.5 mo; RR 31.5%	Selection bias Non-uniform therapeutic regimens, dosing, and scheduling
GOG-0218 [91,108]	1873	Phase III multicenter, randomized, double-blind, PL-controlled	a/m OC 1L, chemotherapy-naïve patients arm 1 (N = 625): 5 cycles carboplatin-paclitaxel + PL followed by 6 cycles of PL alone arm 2 (N = 625): 5 cycles carboplatin-paclitaxel + Bev 15 mg/kgc q3wk followed by 6 cycles of PL alone arm 3 (N = 623): 5 cycles carboplatin-paclitaxel + Bev 15 mg/kgc q3wk followed by 6 cycles of Bev 15 mg/kgc alone	No significant OS or RR benefits were observed in Bev arms. PFS: an increase of PFS was observed in arm 2 (11.6 vs. 10.6 mo; HR 0.89, CI95% 0.78–1.02, *p* = 0.0437) and in arm 3 (14.7 vs. 10.6 mo; HR 0.70, CI95% 0.61–0.81, *p* < 0.0001)	NS
ICON7 (BO17707) [52,91]	1528	Phase III, multicenter, randomized, controlled, open-label	a/m OC following surgery, chemotherapy-naïve patients arm 1 (N = 764): carboplatin-paclitaxel arm 2 (N = 764): same chemotherapy + Bev 7.5 mg/kgc q3wk for up to 12 mo (Bev was initiated at cycle 2 of chemotherapy and within 4 weeks of surgery)	No significant OS benefits were observed in Bev arm. PFS: addition of Bev increased PFS from 16.9 to 19.3 mo (HR 0.86, CI95% 0.75–0.98, *p* = 0.0185) RR: addition of Bev increased RR from 54.9% to 64.7% (*p* = 0.0188)	NS

N—number of subjects; m—metastatic; a—advanced; r—recurrent; CRC—colorectal carcinoma; 1L—first line of therapy; 2+ lines—all subsequent lines of therapy; 2nd line—second-line therapy; mo—months; PL—placebo; IFL—chemotherapy regimen containing 5—Fluorouracil, Irinotecan, and Leucovorin; Bev—Bevacizumab; q2wk—every 2 weeks; q3wk—every 3 weeks; FL—chemotherapy regimen containing 5—Fluorouracil and Leucovorin; OS—overall survival; PFS—progression-free survival; RR—therapy response rate; QoL—quality of life; XELOX—chemotherapy regimen containing oral capecitabine and intravenous oxaliplatin; FOLFOX-4—chemotherapy regimen containing leucovorin plus 5—fluorouracil bolus, followed by 5—fluorouracil infusion, with intravenous oxaliplatin; NS—not specified; BC—breast cancer; NSCLC—non-small-cell lung cancer; OC—ovarian cancer; HR—hazard ratio.

## 5. Conclusions

Bevacizumab remains a high-cost therapy, but it can add to clinical benefits (like overall survival, progression-free survival, and response rate) when used in conjunction with standard chemotherapy in solid tumors. Similar results as those presented in various controlled trials are observable even on unselected cohorts of patients in the uncontrolled conditions of “real-world” oncological practice, thus proving the usefulness of Bevacizumab in solid tumor treatment. Off-label usage is encountered in clinical practice, and this aspect should be monitored given the potential adverse effects of the therapy.

## Figures and Tables

**Table 1:** Sociodemographic characteristics of patients and treatment indication.

**Gender**	Male	238 (38.08%)
	Female	387 (61.92%)
**Age**	<65 years	234 (37.44%)
57.6 years (range 21–85)	≥65 years	391 (62.56%)
**Provenience**	Urban	369 (59.04%)
	Rural	256 (40.96%)
**Comorbidities**	Hypertension	227 (36.32%)
	Diabetes	90 (14.40%)
	Chronic pulmonary disease	43 (6.88%)
	Other cancers	7 (1.12%)
Treatment episodes per tumor origin *	CRC	386 (58.75%)
	NSCLC	47 (7.15%)
	BC	53 (8.07%)
	OC	132 (20.09%)
	CC	31 (4.72%)
	Others	8 (1.22%)
Metastatic site(s) **	Liver	391 (62.56%)
	Lung	344 (55.04%)
	Peritoneum	156 (24.96%)
	Bone	43 (6.88%)
	CNS	32 (5.12%)
	Other	64 (10.24%)

CRC—colorectal cancer; NSCLC—non-small-cell lung cancer; BC—breast cancer; OC—ovarian cancer; CC—cervical cancer; CNS—central nervous system; * patients could receive more than one Bevacizumab-based treatment episode; ** patients may have had more than one metastatic site, and those are counted separately.

**Table 2:** Effectiveness of Bevacizumab-based therapies by type of tumor and treatment line.

	ORR (PR and CR)		Clinical Benefits	PFS		OS	
	N (%)	CI 95%	N (%)	Median (Months)	CI 95%	Median (Months)	CI 95%
**Colorectal (*n* = 379)**							
1L (*n* = 294)	179 (60.9)	42.9–68.9	185 (62.9)	13.5	8.6–18.6	26.3	9.1–43.5
2+ lines (*n* = 85)	23 (27.1)	16.5–41.6	68 (80.0)	6.2	4.7–7.7	9.3	7.7–10.9
**Ovarian (*n* = 127)**							
2+ lines	42 (31.5%)	14.4–46.1	76 (59.8)	7.0	1.3–12.7	11.5	6.0–17.0
**Breast (*n* = 51)**							
1L (*n* = 23)	15 (65.2)	51.1–79.3	19 (82.6)	10.2	6.1–14.3	19.7	16.0–23.4
2+ lines (*n* = 28)	17 (60.7)	46.2–73.8	23 (82.1)	8.1	5.6–10.6	15.6	12.5–18.7
**NSCLC (*n* = 32)**							
1L (*n* = 27)	18 (66.7)	48.1–80.9	20 (74.1)	7.4	6.0–8.9	12.6	8.8–16.4
2+ lines (*n* = 5)	3 (60)	23.0–88.0	4 (80.0)	8.4	3.7–13.1	13.1	0.1–26.2
**Others (*n* = 21)**							
2+ lines	13 (61.9)	32.5–91.3	21 (100)	11.2	2.3–20.1	19.7	4.0–35.3

1L—first line; 2+ lines—second or subsequent treatment lines; NSCLC—non-small-cell lung cancer; ORR—overall response rate (all patients with noticeable shrinkage of tumor(s) either assessed by clinical examination or by imaging measuring); PR—partial response (shrinkage of tumor upon clinical/imaging examination after therapy, but with evident residual tumor); CR—complete response (no clinical/imaging evidence of tumor after therapy); PFS—progression-free survival (the time between the start of therapy and first record of disease progression either assessed by clinician or proved by clinical investigations including imaging); OS—overall survival (the time between the start of therapy and recorded death of patient or censoring); N—number; CI—clearance interval.

**Table 3:** Summary of Bevacizumab-induced adverse effects *.

AE of All Grades (>5% of Patients)	N (%)	Severe AE Leading to Bevacizumab Discontinuation	N
Hemorrhage	167 (25.42)	Hemorrhage	13
Hypertension	42 (6.39)	Hypertension	12
Proteinuria	208 (31.66)	Proteinuria	16
Thromboembolic events	37 (5.63)	Thrombosis/Embolism	9
Abdominal pain	53 (8.06)	Gastrointestinal perforation	10
Nausea/Vomiting	56 (8.52)	Other perforations/fistular complications	4
Fatigue	84 (12.78)	Infusion reaction	1
Septic complications	62 (9.43)	Severe thrombocytopenia	7
Diarrhea	46 (7.00)	Major cardiovascular events	5
		Voluntary withdrawal	2
		Wound-healing deficiencies	2

AE—adverse events; N—number; (%)—frequency; * multiple adverse events appearing in the same patients were counted separately.

## Data Availability

Since the data collected contains personal identifiable information of actual patients, specific questions should be addressed to corresponding authors.
